# Nisin Influence on the Antimicrobial Resistance Ability of Canine Oral Enterococci

**DOI:** 10.3390/antibiotics9120890

**Published:** 2020-12-10

**Authors:** Eva Cunha, Rita Janela, Margarida Costa, Luís Tavares, Ana Salomé Veiga, Manuela Oliveira

**Affiliations:** 1 CIISA—Centro de Investigação Interdisciplinar em Sanidade Animal, Faculdade de Medicina Veterinária, Universidade de Lisboa, Av. da Universidade Técnica, 1300-477 Lisboa, Portugal; evacunha@fmv.ulisboa.pt (E.C.); ritajanela@msn.com (R.J.); margaridaxavicosta@hotmail.com (M.C.); ltavares@fmv.ulisboa.pt (L.T.); 2 Instituto de Medicina Molecular, Faculdade de Medicina, Universidade de Lisboa, Avenida Professor Egas Moniz, 1649-028 Lisboa, Portugal; aveiga@medicina.ulisboa.pt

**Keywords:** nisin, mutant prevention concentration, mutant selection window, antimicrobial susceptibility testing, horizontal gene transfer

## Abstract

Periodontal disease (PD) is one of the most common diseases in dogs. Although previous studies have shown the potential of the antimicrobial peptide nisin for PD control, there is no information regarding its influence in the development of antimicrobial resistance or horizontal gene transfer (HGT). Nisin’s mutant prevention concentration (MPC) and selection window (MSW) were determined for a collection of canine oral enterococci. Isolates recovered after the determination of the MPC values were characterized for their antimicrobial profile and its nisin minimum inhibitory and bactericidal concentrations. The potential of *vanA* HGT between *Enterococcus faecium* CCGU36804 and nine clinical canine staphylococci and enterococci was evaluated. Nisin MPC values ranged from 400 to more than 600 μg/mL. In comparison with the original enterococci collection, the isolates recovered after the determination of the nisin MPC showed increased resistance towards amoxicillin/clavulanate (5%), vancomycin (5%), enrofloxacin (10%), gentamicin (10%) and imipenem (15%). The HGT of *vanA* gene was not observed. This work showed that nisin selective pressure may induce changes in the bacteria’s antimicrobial resistance profile but does not influence horizontal transfer of *vanA* gene. To our knowledge, this is the first report of nisin’s MPC and MSW determination regarding canine enterococci.

## 1. Introduction

Periodontal disease is one of the most widespread inflammatory diseases in dogs [1,2], that results from the establishment of a polymicrobial biofilm (dental plaque) on the teeth surface and a subsequent local inflammatory response [3]. Recently, we proposed the use of a new nisin biogel as a promising strategy to control this disease [4]. Nisin is an antimicrobial peptide, active mainly against Gram-positive bacteria, including multi-drug-resistant bacteria [4,5,6,7], with demonstrated potential for medical application [8]. However, considering that antimicrobial resistance is a major public health problem, any antimicrobial compound under investigation for clinical purposes should be characterized for the mechanisms responsible for resistance development and its environmental persistence [9,10]. Bacteria can become resistant to antimicrobial compounds by acquisition of resistance genes through horizontal gene transfer (HGT), but resistance can also result from the accumulation of mutations that decrease susceptibility [11,12]. Therapeutic protocols that favor mutant subpopulations may facilitate resistance development when compared with regimens that suppress mutant formation [11]. Thus, it is important to optimize the antimicrobial concentrations needed to prevent the selection and amplification of resistant mutants [13]. In this context, the mutant selection window (MSW) hypothesis, described by Zhao and Drlica, postulates that single-step resistant mutant subpopulations, although naturally present, are selectively enriched and amplified when drug concentrations fall within a specific range [14,15,16]. The MSW comprises a range of concentrations between the minimal inhibitory concentration (MIC) and the mutant prevention concentration (MPC) [11]. The MIC is the lowest drug concentration that inhibits the multiplication of the majority of susceptible cells, while MPC is the drug concentration that blocks the growth of the least susceptible, first step mutant, when a high inoculum is applied [11,14,15,16,17,18,19].

As described, resistance dissemination can also occur by HGT, which plays an important role in the emergence of new pathogens [12,20]. The dental plaque biofilm is a perfect environment for the transfer of resistance and virulence genes between bacteria [21]. Present in the oral cavity of dogs with PD, commensal enterococci have a high genome plasticity, being capable of acquiring, conserving, and disseminating genetic determinants, such as resistance genes, easily becoming opportunistic pathogens and being associated with PD-systemic consequences [22,23]. In fact, vancomycin resistance associated with the *vanA* gene is one of the most important antimicrobial resistance determinants associated with vancomycin-resistant *Enterococcus faecium*, considered by WHO as a high priority pathogen [23,24]. This gene is usually present in the Tn1546 transposon, harbored in a plasmid, being transferred by HGT [23,25,26]. Several studies demonstrated that vancomycin resistant enterococci (VRE) can transfer *vanA* to other bacteria, such as staphylococci, which are commensals of the skin and mucosa of animals and humans [23,26]. This transfer ability was associated by some authors to the presence of a pSK41-like plasmids in the recipients [27,28]. Furthermore, a continuous antimicrobial pressure due to the presence of sub inhibitory concentrations of antibiotics in the environment may contribute to the mobilization of acquired resistance genes, being essential to understand how the application of new antimicrobial compounds, such as antimicrobial peptides, can interfere with this phenomenon [26,29,30].

In this work, we determined the MSW of nisin from a previously characterized collection of enterococci obtained from the oral cavity of dogs with PD [31]. The isolates recovered after the determination of the MPC were collected and used to re-evaluate nisin’s inhibitory (MIC) and bactericidal (MBC) concentrations, as well as their antimicrobial susceptibility profile against 11 antimicrobials relevant for veterinary medicine and public health. The influence of subinhibitory concentrations of nisin in the horizontal transfer of *vanA* gene from *Enterococcus faecium* to canine staphylococci and enterococci was also evaluated.

## 2. Results

### 2.1. Determination of the Nisin Mutant Prevention Concentration (MPC)

Nisin MPC values were determined regarding a previously characterized canine enterococci collection, obtained from the oral cavity of dogs with PD [31]. These values, in combination with the previously determined MIC values [1], allowed us to define the MSW of nisin towards the isolates under study. In addition, the relationship between these values, expressed as the ratio MPC/MIC, was determined, which can be used to compare antimicrobial agents for their ability to select resistant mutants [14].

It was possible to determine the MPC values for 85% (*n* = 17) of the strains used, with the exception of strains B28d, B29c and B32a, which presented an MPC higher than 600 µg/mL. The nisin MPC values for the 17 strains ranged from 400 to 600 µg/mL, with an average MPC of 447.06 ± 84.84 µg/mL (Table 1). Considering the nisin MPC/MIC, the resulting MPC values were 15 to 39 times higher than the previously determined MIC values [4].

### 2.2. Antimicrobial Susceptibility Testing

The antimicrobial susceptibility profiles of the original enterococci collection were compared with the ones obtained with the bacterial isolates recovered from the MPC protocol plates with the highest nisin concentrations. It was possible to observe that none of the isolates were susceptible to all the antibiotics tested, being in fact resistant to more than one compound. In the original enterococci collection, resistance levels ranged from 0% (imipenem and amoxicillin/clavulanate) to 100% (cefotaxime and gentamicin-10 μg), while for the isolates recovered from the MPC protocol, the resistance levels varied between 5% (amoxicillin/clavulanate) and 100% (cefotaxime and gentamicin−10 μg). When compared with the original isolates, the MPC recovered isolates presented an increased resistance towards amoxicillin/clavulanate (5%), vancomycin (5%), imipenem (15%), enrofloxacin (10%) and gentamicin-120 µg (10%) (Table 2).

According to the definitions proposed by Magiorakos and collaborators (2012) [32], which indicate that a multidrug-resistant *Enterococcus* spp. is non-susceptible to at least one agent in three or more antimicrobial categories, in our study, 15 isolates (75%) in the original collection and 18 isolates (90%) in the MPC recovered collection exhibited a multidrug-resistance profile.

### 2.3. Determination of Nisin’s Minimum Inhibitory Concentration (MIC) and Minimum Bactericidal Concentration (MBC)

The isolates recovered after determination of the nisin MPC values were used to evaluate the effect of nisin selective pressure on the nisin ’s MIC and MBC values, by comparison with the values from the original collection [1].

MIC values of nisin regarding the isolates of the original collection and the isolates recovered in the MPC protocol are presented in Table 3.

Concerning the isolates recovered after the determination of the MPC, nisin MIC values ranged from 18.75 to 81.25 μg/mL, with an average value of 48.41 ± 21.62 μg/mL. MIC values were higher than 100 μg/mL for three isolates. MBC values ranged from 37.50 to 92.19 μg/mL, with an average value of 60.46 ± 19.40 μg/mL. An MBC value higher than 100 μg/mL was observed for eight isolates.

Nisin MIC results obtained against the MPC recovered isolates were higher and statistically different (*p*-value < 0.05) when compared with the nisin MIC values of the original collection. Concerning the MBC values, no statistical difference was observed between the results regarding the isolates from the two collections; however, most isolates recovered in the MPC protocol showed higher MBC values when compared with the original ones (65%, *n* = 13/20).

### 2.4. Nisin’s Influence on vanA Horizontal Gene Transfer (HGT)

Bacterial acquired resistance by HGT is an important form of resistance dissemination [12]. The *vanA* gene is responsible for vancomycin resistance and its transfer between enterococci and staphylococci is well documented [25]. To evaluate nisin’s influence in *vanA* transfer between enterococci and staphylococci, first we assessed the presence or absence of *vanA* in the isolates under study. In the case of staphylococci, we also evaluated the presence of *mecA* gene, associated with methicillin resistance, and of the pSK41-like plasmid, that may prompt *vanA* transfer [23,28].

In the initial PCR screening, none of the isolates from the oral enterococci collection (*n* = 20) presented the *vanA* gene. In addition, none of the six staphylococci obtained from canine skin lesions presented the *vanA* and *mecA* genes or the pSK41-like plasmid. Then, three isolates from the enterococci collection, M3b, M23a and M29b, were selected to participate in the HGT protocol, based on their strong capacity of biofilm production [31].

Afterwards, two mating rounds aiming to promote HGT of the *vanA* gene from *E. faecium* CCUG 36804 to nine clinical enterococci and staphylococci were performed. One round was performed in the absence of antimicrobial environmental pressure, and another in the presence of subinhibitory concentrations of nisin. None of the two mating experiments allowed the development of transconjugants in the MSA plates supplemented with rifampicin and vancomycin. All isolates recovered from the SBA and the MSA plates supplemented with rifampicin were submitted to PCR analysis, the results of which confirmed the absence of the *vanA* gene.

## 3. Discussion

Antimicrobial resistance is considered one of the major health treats of our time [10,24]. Misuse, overuse, and improper antimicrobial dosage promote a selective environmental pressure to bacteria, favoring resistance development [9,10]. Nisin, commonly used as a preservative in the food industry, is showing relevance in the biomedical field, being a promising antimicrobial agent to be used for the control of canine PD [4,33]. Despite the low resistance rate associated with this antimicrobial peptide, a few cases of nisin resistance have been reported, reinforcing the need to unveil related mechanisms, to evaluate its influence on dental plaque bacterial interaction, and to adopt correct doses to prevent the emergence and amplification of nisin resistant strains [8,34,35].

The MSW hypothesis allows us to define a range of concentrations that can promote mutant’s development, which is useful to evaluate dose regimens [14]. In the present work, the MPC values of nisin against a collection of enterococci from the oral cavity of dogs with PD were determined. The values obtained were up to 39 times higher than the previously determined MIC values for the same isolates [4]. Similar results were also obtained for daptomycin against *Enterococcus faecalis*, with MPC values being 2 to 32 times the MIC values [19]. In fact, several authors have used this methodology to evaluate multiple antimicrobials and bacteria [36,37,38,39,40,41,42,43]. All these studies revealed high MPC values in comparison with the MIC values for the same microorganism; however, a high variation was observed between bacteria and drugs. For example, a MPC/MIC ratio of 48 to 72 was obtained for fosfomycin against *Escherichia coli,* while for *Pseudomonas aeruginosa* the ratio was 28 to 57; likewise, orbifloxacin presented a MPC/MIC ratio against *E. coli* of 4 to 32, while for *P. aeruginosa* the ratio was 16 to 64 [41,43]. In fact, Gianvecchio and collaborators (2019) suggested that MPC values present high variability for a given bacterial strain–antimicrobial combination, and should be understood as a range with confidence intervals, contrasting with MIC values [43,44].

To better understand the effect of nisin selective pressure over 72 h, as promoted in the determination of the MPC, antimicrobial susceptibility profiling along with nisin’s MIC and MBC determination were performed on the isolates obtained in the MPC protocol and results were compared with the original collection.

Considering isolates’ antimicrobial susceptibility profile, differences in the resistance profile of the isolates recovered after MPC protocol were observed when compared with those of the original isolates, specifically concerning amoxicillin/clavulanate, vancomycin, imipenem, enrofloxacin and gentamycin (120 µg). These results suggest a possible influence of nisin in increasing antimicrobial resistance. Cross resistance between nisin and antimicrobials is rare; however, there are some reports describing its occurrence [34,35,45]. Cross resistance may occur regarding antimicrobials that present a similar mode of action, or when the resistance mechanisms are related [35]. Nisin acts by binding to the lipid II, present in the bacterial membrane, which leads to pore formation and inhibition of peptidoglycan synthesis [8]. Considering that, parallel mechanisms may be observed in resistance to vancomycin, an antimicrobial that also acts on lipid II but in a different location, or in resistance to antimicrobials that act on the bacterial wall, such carbapenems or aminopenicillins [5,46]. Resistance to nisin is usually related to proteolytic degradation (by nisinase and nisin resistant protein); however, there are descriptions suggesting that resistance can also arise from mutations that induce changes in the membrane and cell wall composition, such as cell wall thickening, increased positive charges, the presence of penicilin binding proteins and modifications of membrane phospholipid and fatty acid composition [8,34,35,47]. Other nisin resistance mechanisms described so far are related to ABC transporters and multiple regulatory networks [34].

In addition, Drlica (2003) showed that the mutants derived from the MPC protocol are expected to develop mechanisms that inactivate the antimicrobial agent, including efflux or degradation systems [11]. These mechanisms may explain the increased resistance towards enrofloxacin and gentamicin, that act by inhibition of nucleic acid and protein synthesis, respectively [12].

Considering the MIC and MBC determinations, MIC values were higher and statistically different (*p*-value ≤ 0.05) towards all the recovered isolates in comparison with those of the original collection, while MBC values were higher regarding 65% of the recovered isolates in comparison with the originals. These results suggest that incubation in the presence of nisin leads to a reduction in the inhibitory activity, in spite of its bactericidal activity being maintained towards most isolates (60%, Table 3). According to Levinson and collaborators (2009), an MBC/MIC ratio lower than four indicates that the antimicrobial agent is bactericidal [48]. As such, nisin presented a bactericidal activity towards 42% of the isolates from the original collection [4]. On the other hand, nisin presented a bactericidal activity against all isolates recovered from the MPC protocol, except for isolates with nisin MBC values higher than 100 µg/mL.

Animals’ oral cavities present a high bacterial concentration and diversity [21]. Located at the teeth surface, dental plaque is a highly complex polymicrobial biofilm where bacteria easily interact and act as reservoirs of transferable resistance genes [21]. *Enterococcus* spp. are known to be a central hub for resistance gene acquisition, conservation, and dissemination [23]. Classified by the WHO as a high priority pathogen, vancomycin-resistant *Enterococcus faecium*, along with other enterococcal species, are opportunistic pathogens frequently associated with nosocomial infections, and capable of transferring relevant genes to other bacterial species such as *Staphylococcus aureus*, *E. coli* and *Listeria* spp. [23,24,26]. In this work, a protocol aiming to promote the horizontal transfer of *vanA* from *E. faecium* to *Staphylococcus* spp. and *Enterococcus faecalis* clinical isolates was established. This gene is linked to vancomycin and teicoplanin resistance in enterococci, being harbored in a mobile genetic element, allowing its transfer to other bacteria [26]. Mating experiments performed in the absence of nisin selective pressure did not allow the transfer of *vanA* gene. Several studies demonstrated that *vanA* transfer may be facilitated by some molecules, such as pheromone-inducible surface proteins, or be related to the presence of specific plasmids, such as *S. aureus* pSK41 [28,49,50,51]. Although two of our enterococci recipients (M3b and M23a) were able to express an aggregation substance—more specifically, a pheromone-inducible surface protein that facilitates conjugative exchange [4,31,49,52]—no transfer occurred. None of the staphylococcal recipients presented the pSK41 plasmid, which may have influenced the results. Nevertheless, *vanA* gene horizontal transfer is a complex process which is not yet fully understood.

It is known that the use of antimicrobials can enhance gene transfer between bacteria [26]. In order to evaluate the influence of nisin in HGT, mating experiments were performed in the presence of this antimicrobial peptide at subinhibitory concentration [4]. None of the recipients presented the *vanA* gene after the mating experiments, reinforcing the potential of nisin to be used in the clinical setting, more precisely in veterinary medicine for canine PD control.

To our knowledge, this is the first report of nisin’s MPC and MSW determination regarding canine enterococci and of its influence on gene transfer between enterococci and staphylococci. This approach is an important step in the development of new antimicrobial compounds, allowing to understand their potential influence in resistance evolution.

## 4. Materials and Methods

### 4.1. Bacterial Collection

A collection of 20 oral enterococci obtained from the oral cavity of dogs diagnosed with PD, previously characterized regarding clonality, antimicrobial resistance and virulence profiles, were used as bacterial models [31]. From these 20 isolates, 17 correspond to strains belonging to the species *Enterococcus faecalis*, and the remaining 3 to *Enterococcus faecium* [4].

For the HGT protocol, one *Staphylococcus aureus* and five *Staphylococcus pseudintermedius* obtained from canine skin lesions and an *Enterococcus faecium* reference strain (CCUG 36804, *vanA* positive) were used.

*Enterococcus faecalis* ATCC^®^ 29212, *Staphylococcus aureus* ATCC^®^ 25293, a *Staphylococcus aureus mecA* positive strain kindly provided by Dr. Birgit Strommenger, Robert Koch Institute, Germany, and a *Staphylococcus aureus* RN4220 pGO1 positive strain kindly provided by Dr. Alex O’Neill, University of Leeds, were included as controls.

### 4.2. Nisin Preparation

A nisin stock solution (1000 µg/mL) was obtained by dissolving 1 g of nisin powder (2.5% purity, 1000 IU/mg, Sigma-Aldrich, St. Louis, MO, USA) in 25 mL of HCl (0.02 M) (Merck, Darmstadt, Germany) [4]. Then, the stock solution was filtered using a 0.22 µm Millipore filter, and serial dilutions were prepared in distilled sterile water. Solutions were kept at 4 °C during the study.

### 4.3. Determination of the Nisin Mutant Prevention Concentration (MPC)

To determine the MPC of nisin against the canine oral enterococci collection [31], a modified version of the protocol described by Sinel and collaborators (2016) was performed [19]. Briefly, each isolate was spread onto three brain heart infusion (BHI) agar plates (VWR, Leuven, Belgium) and incubated for 24 h at 37 °C. Afterwards, all the bacterial lawn developed in the three BHI plates was resuspended in 450 μL of BHI broth and further incubated at 37 °C for 20 min, to achieve a bacterial suspension of 10^10^ CFU/mL, which was confirmed by viable cell count. Then, an aliquot of 50 μL of this bacterial suspension was inoculated onto Mueller–Hinton (MH) agar plates (Oxoid, Hampshire, UK), supplemented with two-fold concentration increments of nisin ranging from 6.25 to 40× the MIC value of 14.9 μg/mL [4]. Thus, the MH agar plates series contained 6.25, 15, 25, 50, 100, 200, 400, 600 µg/mL of nisin, previously determined. Plates were incubated at 37 °C for 72 h and observed daily for detection of colony growth.

MPC was defined as the lowest concentration of nisin that prevented the growth of any resistant mutant subpopulations after a 72 h incubation period [19]. It was also possible to establish the mutant selection window (MSW) of nisin for the collection of oral enterococci isolates from dogs, a value defined as the antimicrobial concentration ranging between the MIC and MPC values [11,15]. In addition, colonies grown in MH plates with the higher nisin concentration were isolated and kept at −80 °C in a solution of buffered peptone water with 20% glycerol. These isolates were classified as MPC recovered isolates and further used for antimicrobial profiling and for the determination of nisin’s MIC and MBC.

### 4.4. Antimicrobial Susceptibility Testing

Antimicrobial susceptibility profiling was performed regarding the original clinical isolates and those recovered after the MPC protocol to determine if incubation in the presence of nisin interferes with the susceptibility profiles. Using the disk diffusion method, the susceptibility profile regarding a total of 11 different antibiotics, (MASTDISCS^®^ AST, Mast Group, Liverpool, UK), presented in Table 4, was determined in accordance with the Clinical and Laboratory Standards Institute (CLSI) guidelines [53,54]. For that, a 0.5 MacFarland bacterial suspension was prepared for each isolate. Afterwards, the inoculum was evenly spread over the surface of a MH agar plate and the disks impregnated with the antimicrobial agents were placed over the surface of the agar plate. Plates were then incubated at 37 °C under aerobic conditions for 18 h or, in the case of vancomycin, 24 h. After incubation, the inhibition zone diameters were measured and compared with the CLSI standard breakpoints established in VETS01-S2 and M100S, allowing to define the antimicrobial profile (resistant, intermediate or susceptible) of each isolate regarding the antimicrobial agents tested [53,54]. Quality control was performed using the reference strain *Staphylococcus aureus* ATCC^®^ 25293.

Antibiotics were selected based on their relevance to veterinary medicine, as well as to public health. Specifically, CN-120 μg and S-300 μg were included to detect high-level aminoglycoside resistance in *Enterococcus* spp., whereas IMI and VA were chosen due to their importance to public health [53,54,55].

### 4.5. Determination of Nisin’s Minimum Inhibitory Concentration (MIC) and Minimum Bactericidal Concentration (MBC)

Nisin MIC determination was performed regarding the isolates recovered after the MPC protocol using the broth microdilution method, to assess their current susceptibility to nisin, as previously described by Cunha and collaborators (2018) [4]. Briefly, a 96-well microplate was filled with 20 μL of nisin solution at different concentrations (final nisin concentrations ranged from 1.25 to 100 µg/mL) and 180 μL of 10^6^ CFU/mL bacterial suspensions of each isolate. A negative control with only tryptic soy broth (TSB) medium (VWR, Leuven, Belgium) and positive controls with bacterial suspensions were also included.

The 96-well microplates were incubated at 37 °C for 24 h, after which bacterial growth was visually assessed in order to determine MIC value. This parameter is defined as the lowest nisin concentration capable of preventing bacterial multiplication in vitro, with no visible growth on the well [4].

Subsequently, after MIC assessment, the MBC was determined. Five microliters of the bacterial suspension from each well with no visible growth were plated onto tryptic soy agar (TSA) plates (VWR, Leuven, Belgium), followed by incubation at 37 °C for 24 h. MBC was defined as the lowest antimicrobial concentration that inhibits bacterial growth after sub-culture of the suspensions on solid unselective media without any antimicrobial agent [6].

These assays were performed in triplicate, on independent days, and 10% of replicates were tested to assure results representability.

### 4.6. Nisin’s Influence on vanA Horizontal Gene Transfer (HGT)

#### 4.6.1. DNA Extraction and Isolates PCR Screening

DNA extraction was performed based on the protocol described by Semedo-Lemsaddek et al. (2016) and Mottola et al. (2016) [22,56]. Then, all canine staphylococci and enterococci were evaluated by multiplex PCR, in order to identify the presence of the gene *vanA* and *mecA* [57]. Two pairs of primers synthesized by STABVIDA^®^ (Lisbon, Portugal), targeting *vanA* (5′ GGGAAAACGACAATTGC 3′ and 3′ GTACAATGCGGCCGTTA 5′) and *mecA* (5′ TCCAGATTACAACTTCACCAGG 3′ and 3′CCACTTCATATCTTGTAACG 5′) were used [56,57]. The PCR mixture had a final volume of 28.5 μL, with 10 μL of Supreme NZYTaq 2× Green Master Mix (NZYtech^®^, Lisbon, Portugal), 0.5 uM of the *vanA* primer, 0.4 uM of the *mecA* primer, 16.88 μL of PCR-grade water and 5 μL of DNA template. PCR amplification was completed using the conditions: initial denaturation at 94 °C for 4 min; 10 cycles involving denaturation at 94 °C for 30 s, annealing at 64 °C for 30 s and elongation at 72 °C for 45 s; 25 cycles involving denaturation at 94 °C for 30 s, annealing at 50 °C for 45 s and elongation at 72 °C for 2 min, and a final extension step at 72 °C for 10 min. Electrophoresis (90 V for 45 min) was performed to evaluate the amplified products, using a 1.5% agarose gel (NZYtech^®^, Lisbon, Portugal) stained with GreenSafe (NZYtech^®^, Lisbon, Portugal). A molecular weight marker, NZYDNA ladder VI (NZYtech^®^, Lisbon, Portugal) was also included. Results were visualized by transillumination.

Two positive control strains, *Staphylococcus aureus* 01-00694 (*mecA* positive) and *Enterococcus faecium* CCUG 36,804 (*vanA* positive), were included [56].

In addition, the presence of the pSK41-like plasmid in the 6 canine staphylococci under study was evaluated by PCR, using a primer targeting the *traE* gene (5′ ACAAATGCGTACTACAGACCCTAAACGA 3′ and 3′GCCCTGCTGTTGCTGTATCCATATT 5′), synthesized by STABVIDA^®^ [28,58].

A PCR mixture composed by 10 μL of Supreme NZYTaq 2× Green Master Mix (NZYtech^®^, Lisbon, Portugal), 0.4 uM of *traE* primer; 39.2 μL of PCR-grade water and 5 μL of DNA template was used. The PCR amplification was completed using the following conditions: initial denaturation at 94 °C for 2 min; 30 cycles involving denaturation at 95 °C for 15 s, annealing at 55 °C for 90 s and elongation at 72 °C for 90 s, and a final extension step at 72 °C for 7 min [28].

An electrophoresis (90 V for 45 min) was performed to evaluate the amplified products, using a 1.5% agarose gel (NZYtech^®^, Lisbon, Portugal) stained with GreenSafe (NZYtech^®^, Lisbon, Portugal). A molecular weight marker, NZYDNA ladder VII (NZYtech^®^, Lisbon, Portugal), was also included. Results were visualized by transillumination.

A positive control strain, *Staphylococcus aureus* RN4220 (pGO1 positive), was included in the PCR amplification protocol [59].

#### 4.6.2. HGT Protocol

To test if selective pressure due to the presence of subinhibitory concentrations of nisin induces HGT, a protocol adapted from Niederhäusern and collaborators (2011) was developed [25]. Mating experiments were performed in two rounds, using the VRE rifampicin susceptible (Van^r^ Rif^s^) *Enterococcus faecium* CCUG 36804 strain, as donor of the *vanA* gene, and as recipients the 6 canine staphylococci from canine skin lesions, and 3 canine *Enterococcus faecalis* isolates from our collection of enterococci from the oral cavity of dogs with PD, selected according to their strong biofilm forming ability. All recipients were susceptible to vancomycin, and rifampicin resistance was induced (Van^s^ Rif^r^) [25], as it is associated with a point mutation rather than to an acquired gene [60]. After performing a 0.5 MacFarland suspension in 0.9% NaCl for each isolate, 500 µL of the donor suspension and 500 µL of the suspension of one of the recipients were inoculated into 5 mL of TSB and incubated at 37 °C for 18 h. After incubation, 1 mL of the dual bacterial suspension was added to 5 mL of TSB and further incubated for 6 h at 37 °C. Afterwards, 2 mL of each dual suspension were inoculated in TSA plates and incubated for 5 h at 37 °C in a slight movement on a shaker, to promote mating. Then, plates were incubated at 37 °C for 24 h, after which the bacterial suspension that remained at the surface of the agar plates was removed and inoculated in 5 mL of TSB. After an incubation period of 12 h at 37 °C, 100 µL of the suspension was inoculated in Mannitol Salt agar (MSA, PanReac AppliChem, Barcelona, Spain) supplemented with rifampicin (64 µg/mL, PanReac AppliChem, Barcelona, Spain) and vancomycin (8 µg/mL, PanReac AppliChem, Barcelona, Spain), to select for transconjugants. If mating occurred, recombinant isolates developed should be resistant to rifampicin and vancomycin. In addition, the suspension was also inoculated in Manitol Salt Agar (MSA, PanReac AppliChem, Barcelona, Spain) and Slanetz and Bartley agar (SBA, PanReac AppliChem, Barcelona, Spain) supplemented only with rifampicin (64 µg/mL).

The second mating round was performed in the presence of nisin, with all the media used being supplemented with nisin at sub-MIC concentration, 7.45 µg/mL for enterococci [4] and 5.63 µg/mL for staphylococci [6]. All recovered isolates and transconjugants recovered from the supplemented media were submitted to a PCR analysis to detect the presence of the *vanA* gene.

### 4.7. Statistical Analysis

Data statistical analysis was carried out using Microsoft Excel 2016^®^. All quantitative data are expressed as means ± standard deviation. Student’s *t*-test was used for statistical analysis of the nisin MIC and MBC values regarding the original collection and the collection recovered after MPC protocol. A confidence interval of 95% was considered, with a *p*-value ≤ 0.05 indicating statistical significance.

## 5. Conclusions

Periodontal disease is a highly prevalent inflammatory disease in dogs, and nisin might be a promising molecule for its control. The study of nisin influence on mutant’s development, antimicrobial signatures and transfer of resistance determinants revealed that this compound can influence isolates antimicrobial profiles. MPC and MSW determinations can be an interesting measure to establish more accurate treatment protocols based on appropriate antimicrobial doses. However, the utility of the MSW in the definition of dose regimens must be demonstrated not only in vitro but also in vivo. In addition, this study showed that nisin did not promote horizontal transfer of the *vanA* gene between the isolates tested, which emphasizes its potential to be used in PD control. To our knowledge, this is the first report of nisin’s MPC and MSW determination regarding canine enterococci, being a relevant step towards its application in both human and veterinary medicine.

## Figures and Tables

**Table 1:** Minimum inhibitory concentration (MIC), mutant prevention concentration (MPC), and MIC/MPC ratio of nisin against the enterococci collection obtained from the oral cavity of dogs with periodontal disease (PD).

Isolates ID	MIC (µg/mL) [1]	MPC (µg/mL)	MPC/MIC Ratio
M2b	12.75	400	31
M2c	15.75	400	25
M3b	14.75	400	27
M3d	15.75	400	25
M4a	21.50	600	28
M4c	26.75	400	15
M15b	19.25	600	31
M15d	15.25	600	39
M21a	12.50	400	32
M21c	16.00	400	25
M23a	12.50	400	32
M23c	12.50	400	32
M25a	12.50	400	32
M25c	12.50	400	32
M28a	10.50	400	38
M28d	8.50	>600	-
M29b	12.50	400	32
M29c	12.50	>600	-
M32a	17.50	>600	-
M32b	16.25	600	37
**Average**	14.90	447.06	32
**SD**	4.10	84.84	-

ID—identification, MIC—minimum inhibitory concentration, MPC—mutant prevention concentration, SD—standard deviation.

**Table 2:** Representation of the resistance levels of the enterococci from the original collection, obtained from the oral cavity of dogs with PD, and the isolates recovered in the MPC protocol, determined by disc diffusion method according to Clinical and Laboratory Standards Institute (CLSI) guidelines.

Antibiotic	Resistant		Intermediate		Susceptible	
	Number of Original Isolates	Number of MPC Recovered Isolates	Number of Original Isolates	Number of MPC Recovered Isolates	Number of Original Isolates	Number of MPC Recovered Isolates
Ampicillin	3	3	0	0	17	17
Amoxicillin/clavulanate	0	1	3	2	17	17
Vancomycin	2	3	9	8	9	9
Imipenem	0	3	6	5	14	12
Cefotaxime	20	20	0	0	0	0
Enrofloxacin	16	18	4	2	0	0
Ciprofloxacin	11	11	9	9	0	0
Tetracycline	19	19	0	1	1	0
Doxycycline	17	17	2	2	1	1
Gentamicin 10 μg	20	20	0	0	0	0
Gentamicin 120 μg	4	6	0	0	16	14
Streptomycin	15	14	0	0	5	6

**Table 3:** MIC, MBC, and MBC/MIC ratio of nisin regarding the enterococci collection recovered after MPC protocol and the original enterococci collection obtained from the oral cavity of dogs with PD.

Isolates ID	MIC (µg/mL)		MBC (µg/mL)		MBC/MIC Ratio	
	MPC Recovered Isolates	Original Isolates [1]	MPC Recovered Isolates	Original Isolates [1]	MPC Recovered Isolates	Original Isolates
M2b	29.17	12.75	45.83	73.00	1.57	5.73
M2c	29.17	15.75	41.67	85.50	1.43	5.43
M3b	39.58	14.75	43.75	60.25	1.11	4.08
M3d	60.42	15.75	>100	82.25	-	5.22
M4a	>100	21.50	>100	98.50	-	4.58
M4c	>100	26.75	>100	>100	-	-
M15b	79.69	19.25	>100	77.00	-	4.00
M15d	>100	15.25	>100	86.50	-	5.67
M21a	76.56	12.50	>100	59.75	-	4.48
M21c	64.06	16.00	90.63	46.25	1.41	2.89
M23a	56.25	12.50	92.19	64.50	1.64	5.16
M23c	70.31	12.50	76.56	54.25	1.09	4.34
M25a	43.75	12.50	75.00	91.25	1.71	7.30
M25c	62.50	12.50	>100	72.25	-	5.78
M28a	81.25	10.50	>100	48.50	-	4.62
M28d	34.38	8.50	51.56	37.50	1.50	4.41
M29b	27.08	12.50	43.75	41.00	1.62	3.28
M29c	18.75	12.50	64.58	39.25	3.44	3.14
M32a	20.83	17.50	37.50	79.25	1.80	4.53
M32b	29.17	16.25	62.50	69.25	2.14	4.26
**Average**	48.41	14.90	60.46	66.63	1.71	4.70
**SD**	21.62	4.10	19.40	18.57	0.62	1.05

ID—identification, MIC—minimum inhibitory concentration, MBC—minimum bactericidal concentration, MPC—mutant prevention concentration, SD—standard deviation.

**Table 4:** Antimicrobial agents used in the antimicrobial susceptibility test, grouped by mechanism of action, class and concentration [46].

Mechanism of Action	Antimicrobial Class	Antimicrobial Drug	Concentration (µg Per Disk)
Inhibition of cell-wall synthesis	Aminopenicillins	Ampicillin (AMP)	10
		Amoxicillin/Clavulanate * (AMX)	30
	Glycopeptides	Vancomycin (VA)	30
	Carbapenems	Imipenem (IMI)	10
	Cephalosporins	Cefotaxime (CTX)	30
Inhibition of nucleic acid synthesis	Fluoroquinolones	Enrofloxacin (ENR)	5
		Ciprofloxacin (CIP)	5
Inhibition of protein synthesis	Tetracyclines	Tetracycline (T)	30
		Doxycycline (DTX)	30
	Aminoglycosides	Gentamicin (CN)	10/120
		Streptomycin (S)	300

* Beta lactamase inhibitor.
